# Morphological and molecular analyses of parasitic barnacles (Crustacea: Cirripedia: Rhizocephala) in Korea: preliminary data for the taxonomy and host ranges of Korean species

**DOI:** 10.7717/peerj.12281

**Published:** 2021-11-12

**Authors:** Jibom Jung, Ryuta Yoshida, Damin Lee, Joong-Ki Park

**Affiliations:** 1 Division of EcoScience, Ewha Womans University, Seoul, South Korea; 2 Tateyama Marine Laboratory, Marine and Coastal Research Center, Ochanomizu University, Tateyama, Chiba, Japan

**Keywords:** Morphology, Phylogenetic analysis, Taxonomy, Parasitic barnacles, Host range, Korean Rhizocephala

## Abstract

Morphological and molecular analyses of Korean rhizocephalan barnacle species were performed to examine their host ranges and taxonomy. Morphological examination and molecular analysis of mtDNA *cox1*, 16S, and nuclear 18S rRNA sequences revealed nine rhizocephalan species from three genera of the two families, Sacculinidae and Polyascidae. Phylogenetic analysis of molecular sequences revealed two new species candidates in the genus *Parasacculina*, and three *Sacculina* species (*S. pilosella*, *S. pinnotherae*, and *S. imberbis*) were transferred to the genus *Parasacculina.* Examination of host ranges revealed higher host specificity and lower infestation rates in Korean rhizocephalan species than rhizocephalans from other geographic regions. This is the first report of the taxonomy, species diversity, and host ranges of Korean parasitic rhizocephalan barnacles based on their morphological and molecular analyses. More information from extensive sampling of parasitic barnacles from a wide range of crustacean host species is necessary to fully understand their taxonomy, prevalence on decapod hosts, and phylogenetic relationships among major rhizocephalan taxa.

## Introduction

The Rhizocephala comprises morphologically highly modified parasitic barnacles that use a wide range of crustaceans (mostly decapods) as their hosts, mostly parasites on decapods. Members of this group have complex life cycles, usually involving a series of pelagic larval stages followed by an endoparasitic interna stage and a reproductive externa stage where many organ systems (*e.g.*, respiratory, digestive, sensory, and excretory systems) degenerate (Høeg, 1992; Øksnebjerg, 2000). In contrast to other crustacean species, they have a very simplified external structure (externa) and lack segmentation and appendages in the parasitic stage (Høeg & Lützen, 1995). Due to the simplified morphology of the externa, previous taxonomic studies of rhizocephalans have been based largely on larval morphology and the fine structure of externa observed from paraffin sectioning (Yoshida et al., 2011; Kobayashi et al., 2018), with further validation through DNA barcoding analysis (Yoshida et al., 2012; Yoshida, Hirose & Hirose, 2014; Høeg et al., 2019; Jung, Yoshida & Kim, 2019). Very recently, Høeg et al. (2019) modified the taxonomic system of Rhizocephala based on molecular phylogenetic analysis of 18S rDNA sequences, and additional molecular-based taxonomy of rhizocephalan barnacles was updated in Chan et al. (2021).

Since the first report of rhizocephalan barnacles (Krüger, 1912; Høeg & Lützen, 1985), northwestern Pacific species have been reported from southeast Russia (Korn et al., 2020), China (Li et al., 2015), Taiwan (Tu, Chan & Jeng, 2009; Yoshida et al., 2012), Japan (Shiino, 1943; Utinomi & Kikuchi, 1966; Nagasawa, Lützen & Kado, 1996), and Korea (Jung, Yoshida & Kim, 2019). Although Jung, Yoshida & Kim (2019) described 10 species of peltogastrid barnacles from 17 hermit crab species in Korea, the species diversity, distribution, taxonomy, and host range of other rhizocephalan parasitic barnacles are still largely unknown in Korea. In this study, we characterized nine rhizocephalan species, including two new cryptic species candidates, based on morphological examination and molecular analyses of mitochondrial (cytochrome c oxidase subunit I and 16S) and nuclear 18S rDNA sequences. In addition to the taxonomic accounts of the Korean species, we also investigated the prevalence of parasitic barnacles in decapod hosts and phylogenetic relationships among rhizocephalan species.

## Methods

We examined the abdomens of 3,262 individuals of 25 Korean decapod host species collected from 16 sampling sites in Korea. In addition, 12 Japanese rhizocephalans from 11 sampling sites were obtained for comparative molecular study (Tables 1, 2). Korean voucher specimens in this study were deposited in the National Institute of Biological Resources (NIBR) and Honam the National Institute of Biological Resources (HNIBR). Japanese voucher specimens in this study were deposited in the Ryukyu University Museum, Fujukan, University of the Ryukyus, Okinawa, Japan (RUMF), and Coastal Beach of Natural History Museum and Institute, Chiba, Japan (CMNH).

**Table 1:** Individual number and infestation rate (%) of Korean decapod species by rhizocephalan parasitic barnacles examined in this study.

**Host decapod species**	**Total number of individuals examined**	**Number of individuals infested**	**Infestation rate**
*Alpheus bisincisus*	16	0	0.0%
*Arcotheres sinensis*	4	2	50.0%
*Eualus sinensis*	16	0	0.0%
*Gaetice depressus*	1400	14	1.0%
*Helicana japonica*	28	0	0.0%
*Hemigrapsus penicillatus*	50	0	0.0%
*Hemigrapsus sanguineus*	114	9	7.9%
*Hemigrapsus takanoi*	145	1	0.7%
*Ilyoplax dentimerosa*	10	0	0.0%
*Ilyoplax pusilla*	57	0	0.0%
*Laomedia astacina*	15	0	0.0%
*Macromedaeus distinguendus*	74	8	10.8%
*Macrophthalmus (Mareotis) japonicus*	45	0	0.0%
*Neotrypaea japonica*	35	0	0.0%
*Pachygrapsus crassipes*	1	1	100.0%
*Pagurus lanuginosus*	65	0	0.0%
*Pagurus minutus*	811	0	0.0%
*Pagurus nigrofascia*	28	0	0.0%
*Palaemon serrifer*	17	0	0.0%
*Parasesarma pictum*	61	0	0.0%
*Pugettia intermedia*	14	1	7.1%
*Scopimera globosa*	25	0	0.0%
*Sestrostoma balssi*	16	0	0.0%
*Stenalpheops anacanthus*	61	0	0.0%
*Upogebia major*	154	2	1.3%
**Total**	**3262**	**38**	**1.2%**

All rhizocephalan specimens were fixed in 95% ethanol and subjected to morphological examination and molecular analysis. For morphological analysis, the externa and mantle were examined using an MZ8 dissection microscope (Leica, Wetzlar, Germany). Photographs were taken with a D200 digital camera (Nikon, Tokyo, Japan). Carapace length (cl) of the host decapod was measured as the length from the tip of the rostrum to the midpoint of the posterior margin of the carapace using a CD6CSX digital caliper (Mitutoyo, Kawasaki, Japan) to the nearest 0.1 mm.

For molecular analysis, the lateral end of the externa tissue of each rhizocephalan specimen was excised for total genomic DNA extraction using the QIAamp DNA Micro Kit (QIAGEN, Hilden, Germany). Universal primers LCO1490 (5′-GGTCAACAAATCATAAAGATATTGG-3′) and HCO2198 (5′-TAAACTTCAGGGTGACCAAAAAATCA-3′) were used to amplify a fragment of mitochondrial cytochrome c oxidase subunit I (*cox1*) (Folmer et al., 1994). To amplify the mitochondrial 16S rDNA gene, 16SH2 (5′-AGATAGAAACCAACCTGG-3′) and 16SL2 (5′-TGCCTGTTTATCAAAAACAT-3′) primers (Schubart, Neigel & Felder, 2000) were used. For PCR amplification of 18S rDNA, 18S-329R (TAATGATCCTTCCGCAGGTT) and 18S-AF (CAGCMGCCGCGGTAATWC) primers were used (Spears, Abele & Kim, 1992). Polymerase chain reaction (PCR) was performed in reaction volumes of 50 µL that included 2 µL DNA template, 5 µL 10 x Ex Taq Buffer, 2 µL of each primer (10 µM), 0.25 µL Go Taq DNA polymerase (Promega, Madison City, WI, USA), 2.5 µL dNTP mix (10 mM), and 35.75 µL distilled H_2_O. PCR amplification was performed using the following steps: 5 min denaturation at 94 °C followed by 35 cycles of 30 s at 94 °C, 1 min at 52 °C, 1 min at 72 °C, and a final extension of 7 min at 72 °C. PCR products were visualized on 1% agarose gels and sequenced with an ABI PRISM 3730xl DNA analyzer (Applied Biosystems, Foster City, CA, USA). Nucleotide sequences of the three gene fragments (mtDNA *cox1*, 16S, and nuclear 18S rDNA) were analyzed and edited using Geneious *v.* 9.1.8 (Kearse et al., 2012) and aligned using ClustalW in the MEGA10 program (Kumar et al., 2018). Nucleotide sequences were deposited in GenBank (mtDNA *cox1*: MZ216468–MZ216513; 16S: MZ215675–MZ215720; 18S rDNA: MZ215557–MZ215602). Forty-six additional rhizocephalan sequences of Sacculinidae and Polyascidae species available in GenBank were downloaded and included in the phylogenetic analyses (Table 2).

**Table 2:** GenBank accession numbers, geographic information, and host species of rhizocephalan species used for phylogenetic analysis in this study.

**Species**	**Host species**	**Location**	**Specimen number**	***cox1*** **GenBank** **accession no**	**16S rDNA GenBank accession no**	**18S rDNA GenBank accession no**
Boschmaella japonica^*^	*Chthamalus challengeri*	Jôgashima, Japan	ZMUC CRU-3877			AY265369
Briarosaccus regalis^*^	*Paralithodes camtschaticus*	Alaska, USA		KR812178	KR812157	
Clistosaccus paguri^*^		Germany		KT208500		
Heterosaccus californicus^*^	*Loxorhynchus grandis*	CA, USA			AY520756	
		Santa Barbara, USA	ZMUC CRU-3875			AY265359
Heterosaccus dollfusi^*^	*Charybdis longicollis*	Mediterranean			FJ481949	
	*Portunus pelagicus*	Sri Lanka		KY030832		
Heterosaccus lunatus^*^	*Charybdis callianassa*	Moreton Bay, Australia		DQ059778	FJ481947	
Heterosaccus papillosus^*^	*Charybdis bimaculata*	Korea			FJ481948	
Loxothylacus panopaei^*^	*Rhithropanopeus harrisii*	Neuse River, USA	ZMUC CRU-3876		FJ481956	AY265364
	xanthoid crabs	USA		HQ848065		
Loxothylacus texanus^*^	xanthoid crabs	USA		HQ848066		
Mycetomorpha vancouverensis^*^		Alaska, USA			MH974513	
Parasacculina beauforti^*^	*Scylla olivacea*	Malaysia		KX426583		
Parasacculina compressa^*^	*Ozius tuberculosus*	Panglao, Philippines			KF561276	
Parasacculina granifera^*^	*Portunus pelagicus*	Moreton Bay, Australia		DQ059779		
*Parasacculina imberbis* comb. nov.	*Pachygrapsus crassipes*	Namhae, Korea	Korea 4	MZ216470	MZ215675	MZ215557
		Shirosaki, Japan*	S2	AB197804		
Parasacculina leptodiae^*^	*Leptodius affinis*	Labrador, Singapore	ZMUC CRU-3870		FJ481952	AY265365
Parasacculina oblonga^*^	*Cyclograpsus intermedius*	Amakusa, Japan	G4028	DQ059780		
		Tomioka, Japan	ZMUC CRU-3871		FJ481953	AY265367
*Parasacculina pilosella* comb. nov.	*Pugettia intermedia*	Sacheon, Korea	VSJAIV0000000010 (Korea 3)		MZ215679	MZ215561
*Parasacculina pinnotherae* comb. nov.	*Arcotheres sinensis*	Imabari, Japan	CMNH-ZC-02762 (Japan 9)	MZ216499	MZ215676	MZ215558
		Busan, Korea	Korea 1	MZ216468	MZ215677	MZ215559
			Korea 2	MZ216469	MZ215678	MZ215560
Parasacculina sinensis^*^	*Leptodius affinis*	Hong Kong, China	ZMUC CRU-3874			AY265360
*Parasacculina shiinoi*	*Upogebia major*	Namhae, Korea	Korea 25	MZ216486		MZ215562
			Korea 26	MZ216487	MZ215680	MZ215563
		Japan*			KF539761	
*Parasacculina* sp. 1	*Macromedaeus distinguendus*	Sacheon, Korea	Korea 15	MZ216480	MZ215683	MZ215566
			Korea 16	MZ216481	MZ215684	MZ215567
			Korea 17	MZ216482	MZ215685	MZ215568
			Korea 20	MZ216511	MZ215686	MZ215569
			Korea 21	MZ216484	MZ215687	MZ215570
		Yeosu, Korea	Korea 14	MZ216479	MZ215682	MZ215565
			Korea 19			
*Parasacculina* sp. 2	*Macromedaeus distinguendus*	Sacheon, Korea	Korea 18	MZ216483	MZ215688	MZ215571
*Parasacculina* sp.	*Guinusia dentipes*	Tateyama, Japan	CMNH-ZC-02756 (Japan 6)	MZ216497	MZ215681	MZ215564
*Parasacculina yatsui*	*Hemigrapsus sanguineus*	Taean, Korea	VSJAIV0000000011 (Korea 36)	MZ216507	MZ215694	MZ215576
		Toga Bay, Japan*			MG822656	
		Tongyeong, Korea	Korea 38		MZ215695	MZ215577
			Korea 39	MZ216509	MZ215696	MZ215578
	*Pachygrapsus crassipes*	Misaki, Japan	CMNH-ZC-02770 (Japan13)	MZ216503	MZ215691	MZ215574
		Tateyama, Japan	CMNH-ZC-02764 (Japan10)	MZ216500	MZ215689	MZ215572
		Katsuura, Japan	CMNH-ZC-02758 (Japan 7)		MZ215692	
		Tateyama, Japan	CMNH-ZC-02760 (Japan 8)	MZ216498	MZ215693	MZ215575
		Shirosaki, Japan*	S13	AB197810		
		Tateyama, Japan	CMNH-ZC-02768 (Japan 12)	MZ216502	MZ215690	MZ215573
Peltogasterella gracilis^*^	*Pagurus filholi*	Gyeongju, Korea	MADBK 160707_039	MK604152		
	*Pagurus pectinatus*	Busan, Korea	MADBK 430103_002		MK604172	
Peltogaster postica^*^	*Pagurus angustus*	Chisi, Taiwan	NMNS-6795-003		AB778096	
	*Pagurus filholi*	Jeju, Korea	MADBK 430102_002	MK604144		
Peltogaster reticulata^*^	*Pagurus minutus*	Namhae, Korea	MADBK 160706_065		MK604167	
		Vostok Bay, Russia		MN193579		
Polyascus gregarius^*^	*Eriocheir japonica *	Maruyama, Japan	ZMUC CRU-3869			AY265363
*Polyascus* cf. *gregarius*	*Hemigrapsus sanguineus*	Namhae, Korea	VSJAIV0000000013 (Korea 27)	MZ216488	MZ215697	MZ215579
			Korea 30	MZ216513	MZ215700	MZ215582
		Sacheon, Korea	Korea 29	MZ216490	MZ215699	MZ215581
		Taean, Korea	VSJAIV0000000012 (Korea 37)	MZ216508	MZ215703	
		Yeosu, Korea	VSJAIV0000000014 (Korea 28)	MZ216489	MZ215698	MZ215580
			Korea 31	MZ216491	MZ215701	MZ215583
	*Hemigrapsus takanoi*	Namhae, Korea	Korea 32	MZ216492	MZ215702	MZ215584
*Polyascus planus*	*Grapsus albolineatus*	Nakagusuku, Okinawa, Japan	RUMF-ZC-7303 (Japan 2)	MZ216494	MZ215705	MZ215586
		Kenting, Taiwan^*^	ZMUC CRU-3872		FJ481954	AY265368
	*Metopograpsus messor*	Nago, Okinawa, Japan	RUMF-ZC-7305 (Japan 3)	MZ216495	MZ215706	MZ215587
		Iriomote, Japan	RUMF-ZC-7309 (Japan 5)	MZ216496	MZ215707	MZ215588
		Nago, Okinawa, Japan	RUMF-ZC-7301 (Japan 1)	MZ216493	MZ215704	MZ215585
Polyascus polygeneus^*^	*Hemigrapsus sanguineus*	Ôyano, Japan	ZMUC CRU-3873			AY265362
Sacculina carcini^*^	*Carcinus maenas*	Gullmar Fjord, Sweden	ZMUC CRU-3867		FJ481957	AY265366
*Sacculina confragosa*	*Gaetice depressus*	Namhae, Korea	Korea 11	MZ216477	MZ215709	MZ215590
			VSJAIV0000000015 (Korea 12)	MZ216478	MZ215710	MZ215591
		Sacheon, Korea	Korea 5	MZ216471	MZ215715	MZ215597
			Korea 6	MZ216472	MZ215716	MZ215598
			Korea 7	MZ216473	MZ215717	MZ215599
			Korea 8	MZ216474	MZ215718	MZ215600
			Korea 9	MZ216475	MZ215719	MZ215601
			Korea 10	MZ216476	MZ215708	MZ215589
			Korea 23	MZ216485	MZ215712	MZ215593
			Korea 24	MZ216512		MZ215594
		Tongyeong, Korea	VSJAIV0000000016 (Korea 33)	MZ216504	MZ215713	MZ215595
			Korea 34	MZ216505	MZ215714	MZ215596
			Korea 35	MZ216506		
		Yeosu, Korea	Korea 13	MZ216510	MZ215711	MZ215592
	Pachygrapsus crassipes^*^	Shirama, Japan	ZMUC CRU-3868			AY265361
		Shirosaki, Japan	S22	AB197803		
Sacculina insueta^*^	*Ptychognathus riedelii*	Kawasan, Philippines			KF561274	
Sacculina upogebiae^*^					KF539762	
Sacculinidae sp.	*Thalamita* sp.	Tateyama, Japan	CMNH-ZC-02766 (Japan 11)	MZ216501	MZ215720	MZ215602
Sesarmaxenos gedehensis^*^	*Sesarmops* sp.	Kawasan, Philippines			KF561270	
Sylon hippolytes^*^				MG313989		

**Notes.**

* sequences derived from GenBank.

Phylogenetic relationships among rhizocephalan species were inferred for each of the three genes using maximum likelihood (ML) analysis and Bayesian inference (BI) implemented in RaxML version 8 (Stamatakis, 2014) and MrBayes v3.2.6 (Ronquist, Huelsenbeck & Teslenko, 2011), respectively. Phylogenetic trees were modified by MEGA 10. Maximum likelihood analyses of *cox1*, 16S, and 18S rDNA sequences were performed based on the Tamura-Nei (TN93) (Tamura & Nei, 1993), general time reversible (Tavaré, 1986), and Kimura 2-parameter (Kimura, 1980) models, respectively, with a gamma distribution (+G) and invariable sites (+I) rate categories based on Bayesian Information Criterion (BIC) scores model using the Model Selection option of MEGA10. The robustness of individual nodes in the ML trees was assessed by analysis of 1,000 bootstrap replications. Interspecific and intraspecific sequence divergences were estimated based on the K2P distance matrix in MEGA10.

## Results

**Figure 1:**
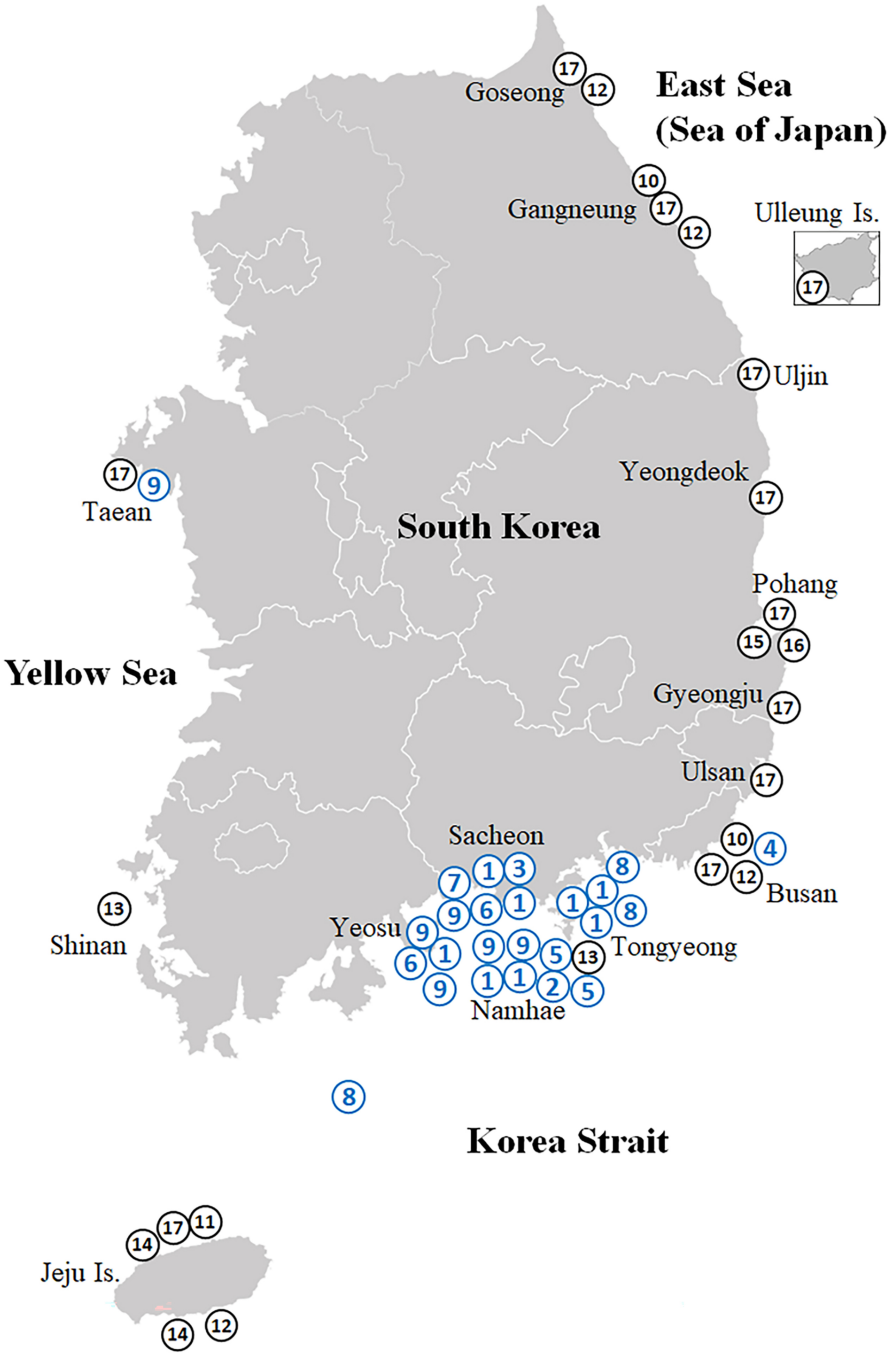
Map showing the collection sites of the Korean rhizocephalan species. Numbered circles indicate sampling localities where rhizocephalan species were sampled in this study (blue) and Jung, Yoshida & Kim (2019); black). 1, *Sacculina confragosa*; 2, *Parasacculina imberbis*; 3, *Parasacculina pilosella*; 4, *Parasacculina pinnotherae*; 5, *Parasacculina shiinoi*; 6, *Parasacculina* sp. 1; 7, *Parasacculina* sp. 2; 8, *Parasacculina yatsui*; 9, *Polyascus* cf. *gregarius*; 10, *Peltogaster lineata*; 11, *Peltogaster postica*; 12, *Peltogaster* aff. *ovalis*; 13, *Peltogaster* aff. *reticulatus*; 14, *Peltogaster* sp. 1; 15, *Peltogaster* sp. 2; 16, *Peltogaster* sp. 3; 17, *Peltogasterella gracilis*.

Based on morphological examination (shape and number of externae and mantle aperture) and mitochondrial sequence information, we identified 38 rhizocephalan individuals belonging to nine species, three genera, and two families isolated from eight decapod hosts species collected from 16 sites (Fig. 1; Table 2). All rhizocephalans identified by this study except *Parasacculina pinnotherae* comb. nov. were first reported from Korea. Detailed information regarding the Korean rhizocephalan species and their externa morphology is provided in Table 3.

### Taxonomic accounts and morphological features of Korean rhizocephalan species

**Table:** 

Sacculinidae Lilljeborg, 1861
*Sacculina* Thompson, 1836

### *Sacculina confragosa* Boschma1933 (Fig. 2A)

Materials examined: on *Gaetice depressus*: 1 ind., Sacheon (34.9 N 128.1 E), Korea 5, host: ♀, cl 11.5 mm; 1 ind. (2 externa), Sacheon (34.9 N 128.1 E), Korea 6, host: ♀, cl 11.8 mm; 1 ind., Sacheon (34.9 N 128.0 E), Korea 7, host: ♀, cl 13.7 mm; 1 ind., Sacheon (34.9 N 128.0 E), Korea 8, host: ♀, cl 9.1 mm; 1 ind., Sacheon (34.9 N 128.1 E), Korea 9, host: ♀, cl 10.3 mm; 1 ind., Sacheon (34.9 N 128.1 E), Korea 10, host: ♀, cl 11.8 mm; 1 ind., Namhae (34.7 N 127.9 E), Korea 11, host: ♂, cl 11.9 mm, feminization; 1 ind. (2 externa), Namhae (34.7 N 127.9 E), VSJAIV0000000015, Korea 12, host: ♀, cl 7.7 mm; 1 ind., Yeosu (34.7 N 127.8 E), Korea 13, host: ♂, cl 11.2 mm, feminization; 1 ind., Sacheon (34.9 N 128.0 E), Korea 23, host: ♂, cl 12.0 mm; 1 ind., Sacheon (34.9 N 128.0 E), Korea 24, host: ♂, cl 13.9 mm; 1 ind., Tongyeong (34.8 N 128.4 E), VSJAIV0000000016, Korea 33, host: ♀, cl 7.1 mm; 1 ind., Tongyeong (34.8 N 128.4 E), Korea 34, host: ♂, cl 14.9 mm; 1 ind., Tongyeong (34.6 N 128.5 E), Korea 35, host: ♂, cl 13.5 mm.

**Table 3:** Morphological features of the externa of nine Korean rhizocephalans.

**Species**	**Externa**		**Mantle aperture**	
	**Shape**	**Externa number**	**Projection**	**Opening**
*Sacculina confragosa*	Wrinkled flat cordiform	Single or double	Elevated tube	Circular
*Parasacculina imberbis* comb. nov.	Smooth round-rectangular	Single		
*Parasacculina pilosella* comb. nov.	Smooth and slightly flat oval	Single	Flat	Circular
*Parasacculina pinnotherae* comb. nov.	Smooth or slightly wrinkled flat oval or cordiform	Single or double	Elevated	Dot shaped
*Parasacculina shiinoi*	Smooth oval	Single		
*Parasacculina* sp. 1	Smooth or slightly wrinkled oval	Single	Elevated	Circular
*Parasacculina* sp. 2	Smooth oval	Single	Slightly elevated	Circular
*Parasacculina yatsui*	Smooth or slightly wrinkled flat oval or flat cordiform	Single	Elevated	Slit shaped
*Polyascus* cf. *gregarius*	Smooth or slightly wrinkled flat cordiform	Single	Elevated	Slit shaped

**Figure 2:**
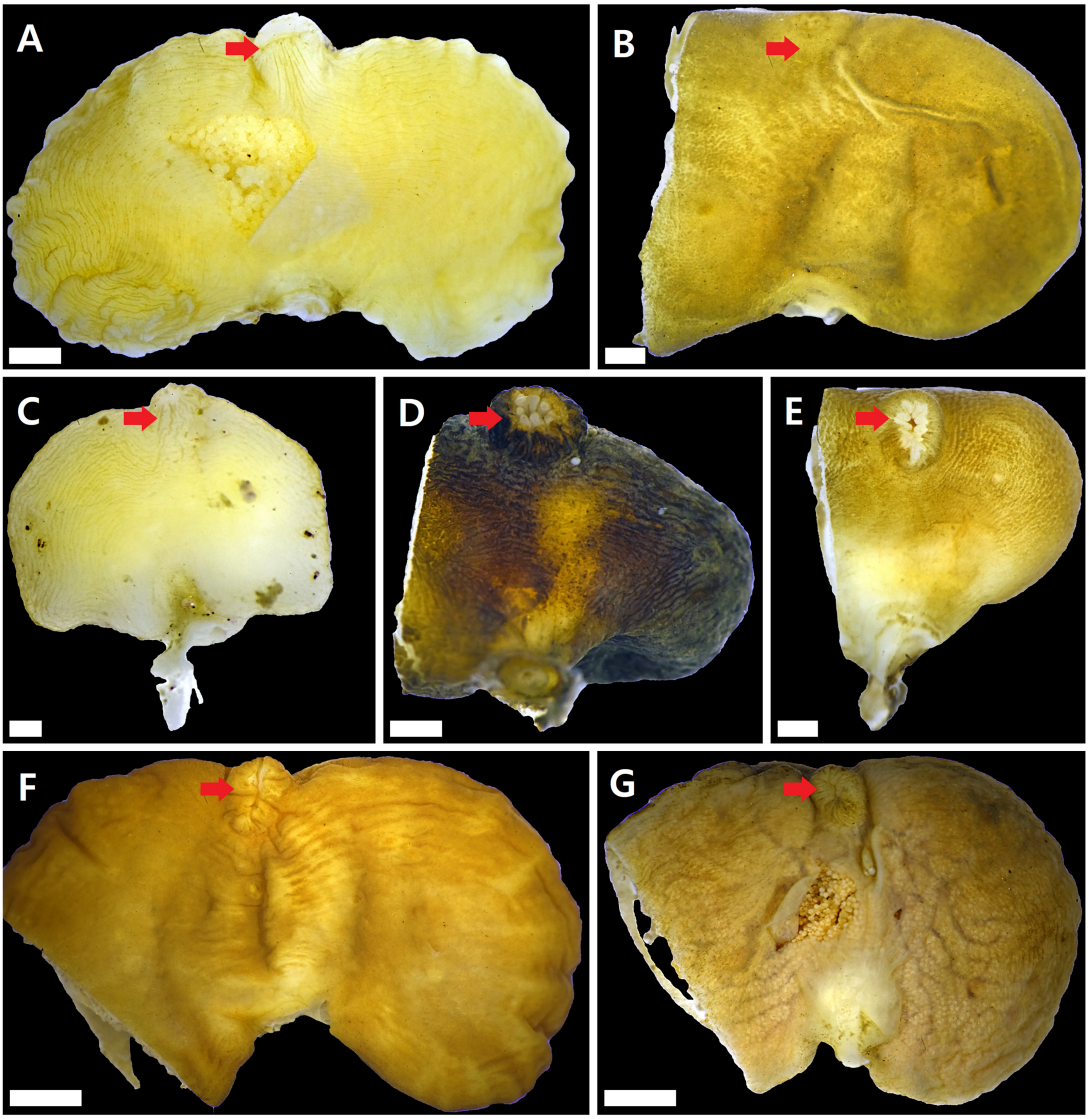
Externae of Korean rhizocephalans. Red arrow: mantle, scale bar: two mm. (A) *Sacculina confragosa.* (B) *Parasacculina pilosella* comb. nov. (C) *Parasacculina pinnotherae* comb. nov. (D) *Parasacculina* sp. 1. (E) *Parasacculina* sp. 2. (F) *Parasacculina yatsui.* (G) *Polyascus* cf*. gregarius.* Externae of some specimens (B, D–G) were incomplete, in case they were used for molecular analysis (B, D–G).

Host species: *G. depressus*, *Pachygrapsus crassipes* (Grapsidae), *Cyclograpsus intermedius* (Varunidae).

Distribution: Japan, Korea.

Diagnosis of the externa: whole externa mostly single and occasionally double, wrinkled cordiform with flat half-oval-shaped left and right lobes divided by an outer mid-groove and inner mid-ridge; outermost part of the robe wrinkled. Mantle well elevated, tube-shaped, and vertically slightly wrinkled with a circular opening at the extremity.

Remarks: Morphological characteristics of the examined materials correspond with their original description (Boschma, 1933) except for the number of externa. Some of our specimens (Korea 6, Korea 12) had double externae (15% of total examined individuals), whereas others had a single externa. This type of variation in the number of externa has been reported in a previous study (Shiino, 1943). This species is found most abundantly parasitizing medium-sized individuals of host crab species. Further study is needed to determine if this species is a predominant parasitic form on medium-sized host individuals.

### *Parasacculina imberbis* (Shiino, 1943) comb. nov.

Polyascidae Høeg & Glenner in Høeg et al., 2019

*Parasacculina* Høeg & Glenner in Høeg et al., 2019

Materials examined: 1 ind., Namhae (34.7 N 128.0 E), Korea 4, host: ♀, cl 12.5 mm.

Host species: *Pachygrapsus crassipes* (Grapsidae).

Distribution: Japan, Korea.

Diagnosis of the externa: whole externa smooth, single, with a rounded-rectangular shape.

**Figure 3:**
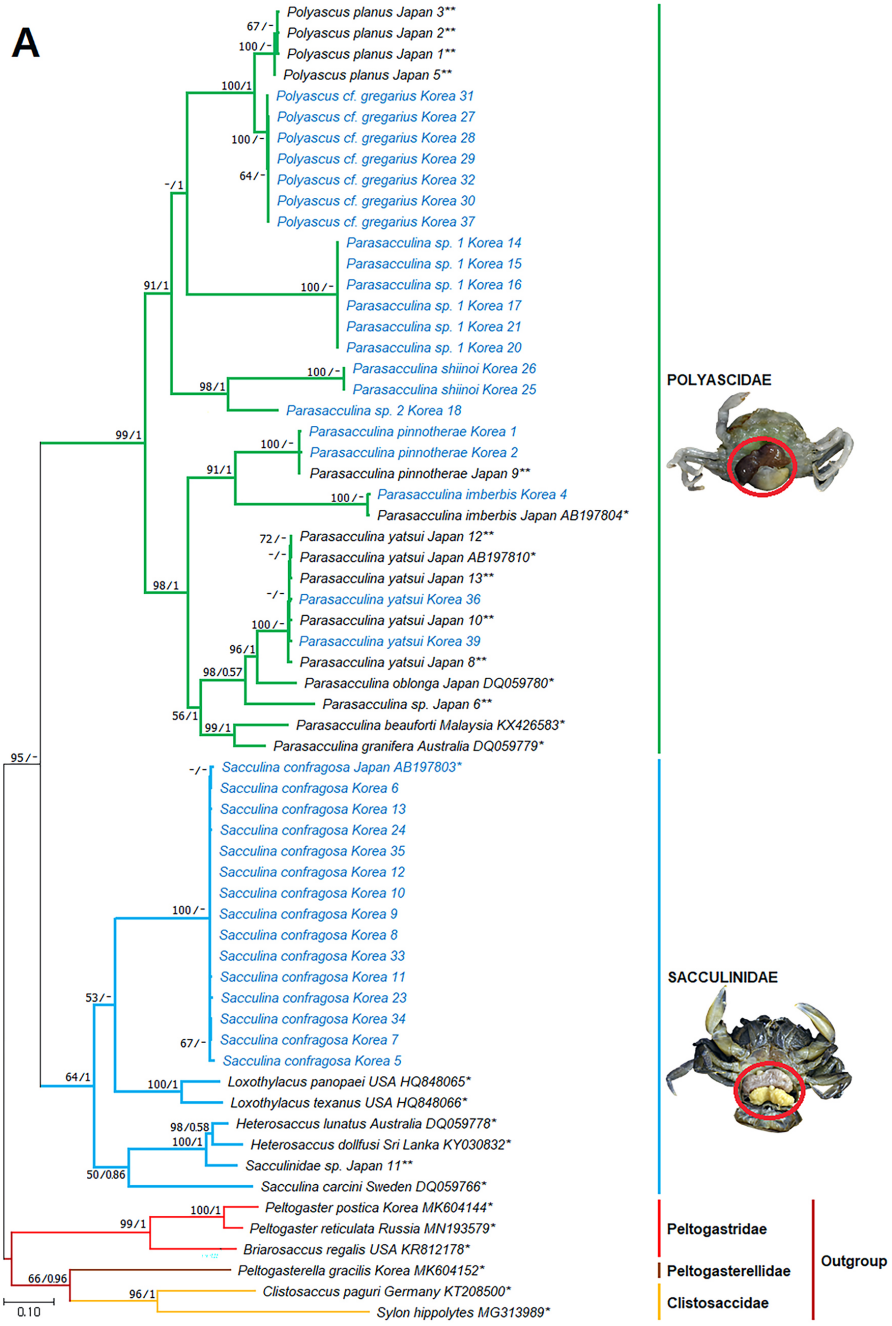
Phylogenetic tree of *cox1* from rDNA from Korean rhizocephalan species using Maximum likelihood and Bayesian inference methods. Values on nodes indicate maximum likelihood bootstrap support/Bayesian posterior probability. Sequences from Korean species determined in this study are indicated in blue. *: sequences derived from GenBank, **: Japanese sequences obtained in this study.

Remarks: The examined specimen had a single externa, but we were not able to examine morphological characteristics in more detail due to the immature stage of the specimen. Species identification of this specimen and its taxonomic placement in the genus *Parasacculina* were based on molecular analyses of mtDNA *cox 1*, 16S, and nuclear 18S gene sequences (Figs. 3A–3C; see Discussion for more details).

**Figure 4:**
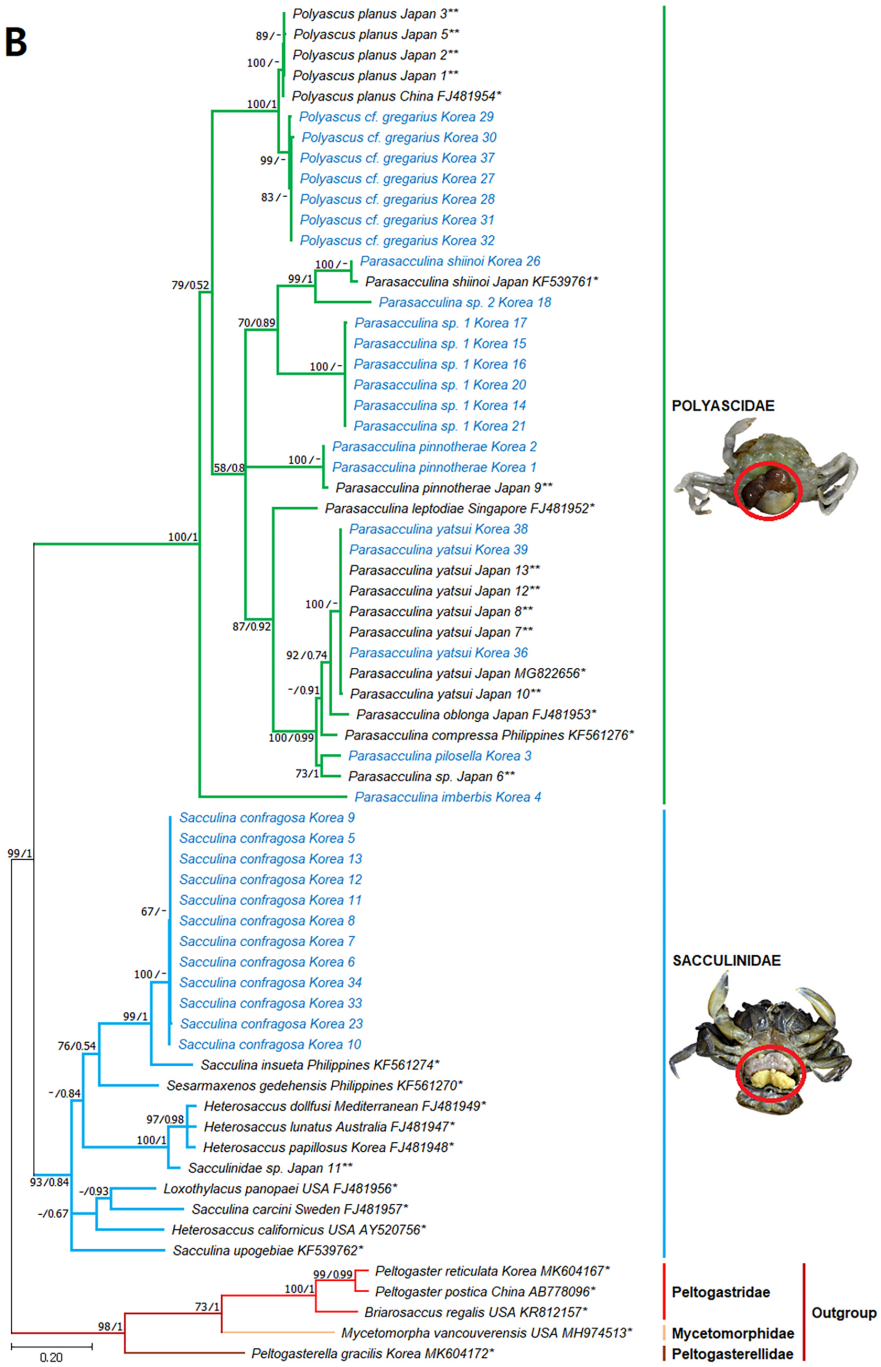
Phylogenetic tree of 16S rDNA from Korean rhizocephalan species using Maximum likelihood and Bayesian inference methods. Values on nodes indicate maximum likelihood bootstrap support/Bayesian posterior probability. Sequences from Korean species determined in this study are indicated in blue. *: sequences derived from GenBank, **: Japanese sequences obtained in this study.

**Figure 5:**
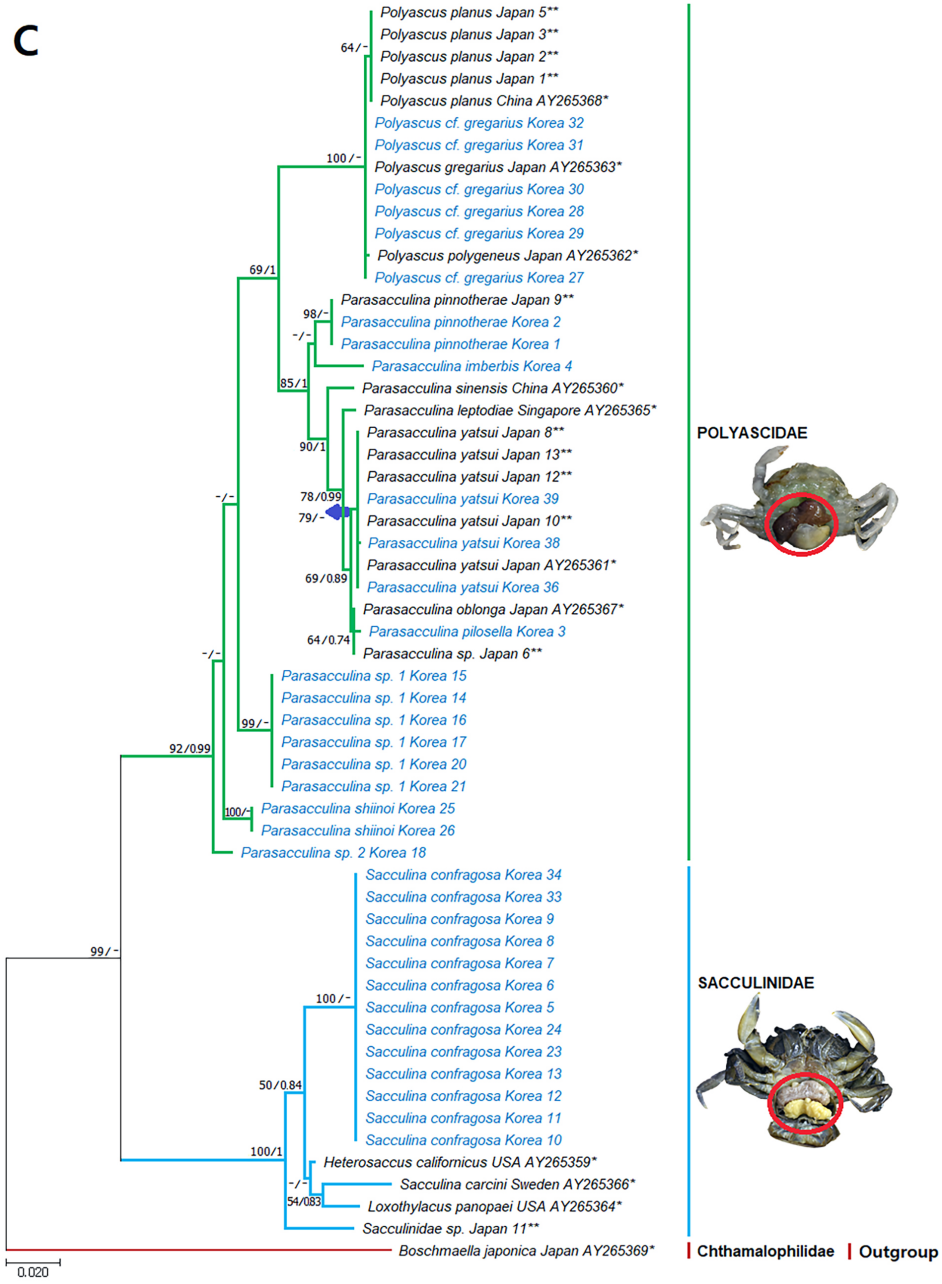
Phylogenetic tree of 18S rDNA from Korean rhizocephalan species using Maximum likelihood and Bayesian inference methods. Values on nodes indicate maximum likelihood bootstrap support/Bayesian posterior probability. Sequences from Korean species determined in this study are indicated in blue. *: sequences derived from GenBank, **: Japanese sequences obtained in this study.

**Figure 6:**
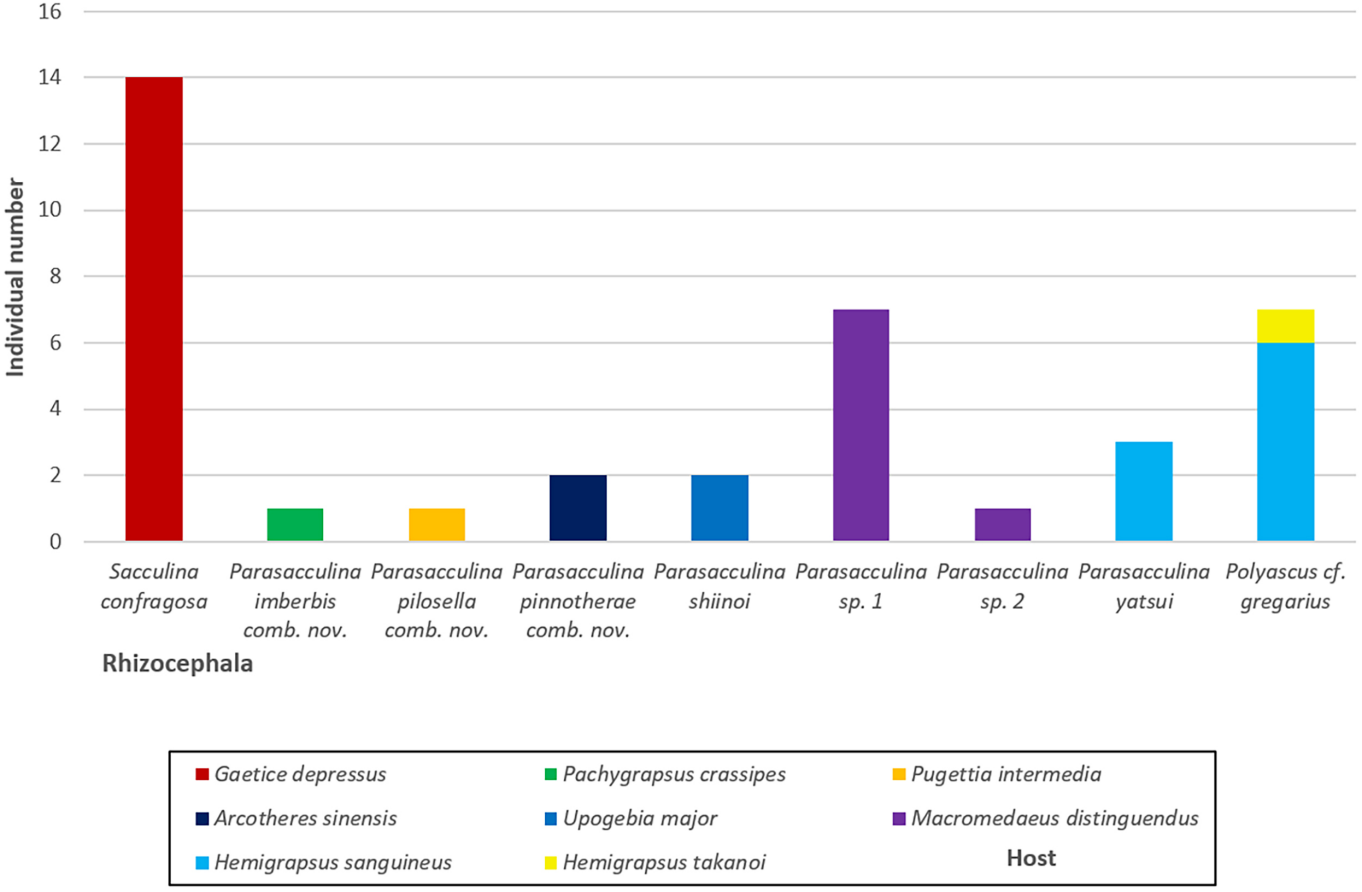
A vertical bar chart showing the individual number of nine Korean rhizocephalan species found from their hosts.

### *Parasacculina pilosella* (Kampen & Boschma, 1925) comb. nov. (Fig. 2B)

Material examined: 1 ind., Sacheon (34.9 N 128.1 E), VSJAIV0000000010, Korea 3, host: ♂, cl 13.7 mm.

Host species: *Pugettia intermedia* (Epialtidae).

Distribution: Indonesia, Japan, Korea.

Diagnosis of the externa: whole externa smooth, single, and slightly flat and oval. Mantle flat and vertically slightly wrinkled with a circular opening at the extremity.

Remarks: Four *Sacculina* species (*S. muricata* Boschma, 1931, *S. pugettiae* (Shiino, 1943), *S. reinhardi* (Boschma, 1955), and *S. pilosella*) were previously reported to parasitize *Pugettia* spp*.*. Morphological characteristics of the examined specimen correspond with the original description of *S. pillosella* (Van Kampen & Boschma, 1925). However, phylogenetic analysis of mtDNA 16S and 18S rDNA sequences showed that this species is nested within *Parasacculina* species (Figs. 3B and 3C), separated from *Sacculina* species (*S. confragosa, S. upogebiae*, and *S. carcini*). Therefore, we consider this species a member of the genus *Parasacculina* (see Discussion for more details).

### *Parasacculina pinnotherae* (Shiino, 1943) comb. nov. (Fig. 2C)

Materials examined: 1 ind., Busan (35.2 N 129.2 E), Korea 1, host: ♀, cl 9.1 mm, in the mussel; 1 ind. (2 externa), Busan (35.2 N 129.2 E), Korea 2, host: ♂, cl 6.9 mm, in the mussel.

Host species: *Arcotheres sinensis* (Pinnotheridae).

Distribution: Japan, Korea.

Diagnosis of the externa: whole externa smooth or slightly wrinkled, single or double, and flat oval or cordiform in shape; each outer-posterior margin elevated into a conical shape. Mantle slightly elevated and vertically wrinkled with a small round opening at the extremity.

Remarks: Two *Sacculina* species (*S. pertenuis* (Boschma, 1933) and *S. pinnotherae*) have been reported to be parasitic on *Pinnotheres* spp*.*. Morphological characters of examined specimens correspond with the original description of *S. pinnotherae* (Shiino, 1943).

However, phylogenetic analysis of mtDNA *cox* 1, 16S, and nuclear 18S rDNA sequences placed this species within the genus *Parasacculina* (Figs. 3A–3C), not in the genus *Sacculina.* Therefore, we treated this species as a member of the genus *Parasacculina* (see Discussion for more details). The host crab (*Arcotheres sinensis*) is known to parasitize bivalves, so *P. pinnotherae* comb. nov. is a secondary parasite that is rare in the ocean (McDermott, 2009).

### *Parasacculina shiinoi* (Lützen et al., 2016)

Materials examined: 1 ind., Namhae (34.9 N 127.9 E), Korea 25, host: cl 8.8 mm; 1 ind., Namhae (34.9 N 127.8 E), Korea 26, host: cl 11.1 mm.

Host species: *Upogebia major* (Upogebiidae).

Distribution: Japan, Korea.

Diagnosis of the externa: whole externa smooth, single, oval in shape.

Remarks: The examined specimens had a single externa, but detailed morphological characteristics could not be determined because of the immaturity of the specimens examined. Lützen et al. (2016) reported that *Sacculina upogebiae* parasitizes *Upogebia* species. Molecular analysis of mtDNA 16S rDNA sequences revealed that this species grouped with *P. shiinoi* (GenBank accession no: KF539761: Fig. 3B) with very high sequence identity (98.9%).

### *Parasacculina* sp. 1 (Fig. 2D)

Materials examined: 1 ind., Yeosu (34.7 N 127.8 E), Korea 14, host: ♀, cl 7.6 mm; 1 ind., Sacheon (34.9 N 128.0 E), Korea 15, host: ♀, cl 8.0 mm; 1 ind., Sacheon (34.9 N 128.0 E), Korea 16, host: ♂, cl 9.7 mm; 1 ind., Sacheon (34.9 N 128.0 E), Korea 17, host: ♂, cl 9.9 mm; 1 ind., Yeosu (34.7 N 127.8 E), Korea 19, host: ♂, cl 14.6 mm; 1 ind., Sacheon (34.9 N 128.0 E), Korea 20, host: ♂, cl 14.1 mm; 1 ind., Sacheon (34.9 N 128.0 E), Korea 21, host: ♂, cl 17.7 mm.

Host species: *Macromedaeus distinguendus* (Xanthidae).

Distribution: Korea.

Diagnosis of the externa: whole externa single, smooth or slightly wrinkled, and oval in shape. Mantle large, elevated, and vertically wrinkled with circular opening at the extremity.

Remarks: *Parasacculina leptodiae* and *P. sinensis* have been reported to be parasites of *Leptodius affinis*, the most phylogenetically similar host species to *M. distinguendus* among the currently known hosts of Rhizocephala. However, the specimens examined in this study differ in morphology and molecular sequences from *P. leptodiae* and *P. sinensis*. This species has a single externa, whereas *P. leptodiae* has multiple externae. In addition, this species has a large, elevated mantle aperture, but *P. leptodiae* and *P. sinensis* have a flat mantle (Guérin-Ganivet, 1911; Boschma, 1933). Phylogenetic analysis clearly showed that the *cox* 1, 16S, and 18S rDNA sequences of this species are different from those of *P. leptodiae* and *P. sinensis* and all other *Parasacculina* species included in the analyses (Figs. 3A–3C). Therefore, we considered this species to be a new species candidate of the genus *Parasacculina* (see Discussion for more details).

### *Parasacculina* sp. 2 (Fig. 2E)

Material examined: 1 ind., Sacheon (34.9 N 128.1 E), Korea 18, host: ♂, cl 13.5 mm.

Host species: *Macromedaeus distinguendus* (Xanthidae).

Distribution: Korea.

Diagnosis of the externa: whole externa smooth, single, and oval in shape. Mantle slightly elevated with circular opening at extremity.

Remarks: Previously, *P. leptodiae* and *P. sinensis* were known to be parasites of *L. affinis,* the most phylogeneically similar host species to *M. distinguendus* among the currently known hosts of Rhizocephala. However, the examined specimen of this species differed in morphology and molecular sequence from all *Parasacculina* species including *Parasacculina* sp. 1. This species has one externa compared to the multiple externae of *P. leptodiae*. In addition, this species has a slightly elevated mantle aperture, whereas *P. leptodiae* and *P. sinensis* have a flat mantle (Guérin-Ganivet, 1911; Boschma, 1933), and *Parasacculina* sp. 1 has a well-elevated mantle. Furthermore, phylogenetic analysis of mtDNA *cox* 1, 16S, and nuclear 18S rDNA sequences distinguished this species from other *Parasacculina* species with 18.1–30.6% sequence divergence in *cox1*, 14.0–27.5% sequence divergence in 16S rDNA, and 1.9−4.2% sequence divergence in 18S rDNA (Figs. 3A–3C). Therefore, we treated this species as a new species candidate of the genus *Parasacculina* (see Discussion for more details).

### *Parasacculina yatsui* (Boschma, 1936) (Fig. 2F)

Materials examined: on *Hemigrapsus sanguineus*: 1 ind., Yeosu (34.1 N 127.3 E), VSJAIV0000000011, Korea 36, host: ♀, cl 22.3 mm; 1 ind., Tongyeong (34.8 N 128.4 E), Korea 38, host: ♂, cl 16.9 mm, feminization; 1 ind., Tongyeong (34.6 N 128.5 E), Korea 39, host: ♂, cl 14.2 mm, feminization.

Host species: *Pachygrapsus crassipes* (Grapsidae), *H. sanguineus* (Varunidae).

Distribution: Japan, Korea.

Diagnosis of the externa: whole externa smooth or slightly wrinkled, single, and cordiform with flat half-oval-shaped left and right lobes divided by outer mid-groove and inner mid-ridge; outermost part of the robe wrinkled. Mantle tube-shaped, elevated, and slightly wrinkled with slit opening at extremity.

### *Polyascus* cf. *gregarius* (Okada & Miyashita, 1935) (Fig. 2G)

*Polyascus* Glenner, Lützen & Takahashi, 2003

Materials examined: on *H. sanguineus*: 1 ind., Namhae (34.7 N 127.9 E), VSJAIV0000000013, Korea 27, host: ♀, cl 18.4 mm; 1 ind., Yeosu (34.7 N 127.8 E), VSJAIV0000000014, Korea 28, host: ♂, cl 29.8 mm, feminization; 1 ind., Sacheon (34.9 N 128.1 E), Korea 29, host: ♀, cl 9.8 mm; 1 ind., Namhae (34.9 N 127.8 E), Korea 30, host: ♂, cl 16.7 mm, feminization; 1 ind., Yeosu (34.7 N 127.8 E), Korea 31, host: ♂, cl 8.9 mm, feminization; 1 ind., Taean (36.8 N 126.1 E), Korea 37, host: ♂, cl 14.1 mm, feminization.

on *H. takanoi*: 1 ind., Namhae (34.9 N 127.9 E), Korea 32, host: ♂, cl 9.5 mm.

Host species: *Hemigrapsus sanguineus, H. takanoi, Eriocheir japonica* (Varunidae).

Distribution: Japan, Korea.

Diagnosis of the externa: whole externa smooth or slightly wrinkled, single, and flat-cordiform shaped with flat half-oval-shaped left and right lobes divided by an outer mid-groove and inner mid-ridge; outermost part of the robe smooth or slightly wrinkled. Mantle tube-shaped, elevated, and vertically wrinkled with slit-shaped opening at the extremity.

Remarks: Morphological characteristics of the examined materials correspond with their original description (Okada & Miyashita, 1935) except for the number of externa and the host species. All specimens examined in this study had a single externa, whereas *P. gregarius* has multiple externae. In addition, the host species (*H. sanguineus* and *H. takanoi*) differ from the host species reported for *P. gregarius*, namely *E. sinensis*. Nevertheless, this species is likely *P. gregarius* because 18S rDNA sequences of these specimens were identical to the GenBank sequences of *P. gregarius* (Fig. 3C). In addition, individual variation in the number of externa of rhizocephalans has also been reported previously (Reinhard, 1942; Shiino, 1943; Høeg & Lützen, 1985).

In phylogenetic trees (Figs. 3A–3C), *Polyascus* cf. *gregarious* was clustered with *P. planus* that is commonly found in Japan and Taiwan. These two species are similar in having a flat-cordiform shaped externa, but different in some aspects of morphology and host species: the former has an elevated mantle and single externa, while the latter has an underdeveloped mantle and multiple externae (Boschma, 1933). In addition, the Varunidae crabs (H. sanguineus and H. takanoi) are used as P. cf. gregarious hosts, whereas the Grapsidae crabs (Grapsus albolineatus and Metopograpsus messor) are known as P. planus host (Tu, Chan & Jeng, 2009). Morphological and host range variation among rhizocephalan species has been reported by previous studies (Høeg & Lützen, 1985; Jung, Yoshida & Kim, 2019), and thus further studies with broader taxon sampling of *P. gregarious* and *P. planus* are needed to confirm an accurate species delimitation in their morphology and host range.

### Phylogenetic relationships among rhizocephalan species

Since only Sacculinidae and Polyascidae species were found in this study, we focused on phylogenetic relationships among rhizocephalan species in these two families. Totals of 34 *cox1* (555 bp), 33 16S rDNA (474 bp), and 35 18S rDNA (1002 bp) sequences were used for phylogenetic analysis, and the resulting ML and Bayesian trees were consistent with each other in that Sacculinidae and Polyascidae were monophyletic (Figs. 3A–3C). In all phylogenetic trees, the sequences of Korean rhizocephalans species nested and/or clustered with sequences of the same species retrieved from GenBank (Figs. 3A–3C).

*Parasacculina* sp. 1 and 2 were recognized as new species candidates because they did not show sister relationships with other *Parasacculina* species (Figs. 3A–3C). In the 16S and 18S DNA trees, they were placed at different positions and separated from *P. leptodiae* and *P. sinensis*, which share the host family and have similar morphological characteristics (Figs. 3B and 3C). *Parasacculina* sp. 1 and *Parasacculina* sp. 2 formed a group with *P. shiinoi,* but their 16S pairwise sequence divergences from *P. shiinoi* were substantial, ranging from 18.2–20.9% for *Parasacculina* sp. 1 and 14.4–14.8% for *Parasacculina* sp. 2. This group (*Parasacculina* sp. 1, *Parasacculina* sp. 2, and *P. shiinoi*) was separate from *P. leptodiae* in the 16S tree, whereas it was basal to the remaining Polyascidae species, including *P. leptodiae* and *P. sinensis*, in the 18S tree. In the *cox1* tree, *Parasacculina* sp. 1 formed a well-defined sister group to *Polyascus* species (*P.* cf. *gregarious* and *P. planus*), whereas *Parasacculina* sp. 2 was grouped with the Korean isolate of *P. shiinoi* showing 18.1% sequence divergence (Fig. 3A). Interspecific sequence differences of the two new species candidates from other Polyascidae species were 18.1–32.1% for *cox1*, 14.0–28.8% for 16S rDNA, and 1.9−4.7% for 18S rDNA. In contrast to the high interspecific sequence divergences discovered, there were no individual variations in *cox1*, 16S, and 18S rDNA sequences among *Parasacculina* sp. 1 specimens.

The three *Sacculina* species (*S. imberbis, S. pilosella*, and *S. pinnotherae*) clustered with *Parasacculina* species (Figs. 3A–3C): *S. imberbis* grouped with *S. pinnotherae,* that is sister to other *Parasacculina* species based on analysis of *cox1* (*P. yatsui, P. granifera*) and 18S rDNA (*P. yatsui, P. sinensis, P. leptodiae*) sequences (Figs. 3A, 3C). *S. pilosella* formed a sister group to *P. compressa*, *P. oblonga*, and *P. yatsui* in the 16S and 18S rDNA trees (Figs. 3B and 3C). Interspecific sequence differences between *Sacculina* species and *Parasacculina* species were remarkably large, ranging from 27.3–33.4% for *cox1*, 27.5–34.9% for 16S rDNA, and 9.2–10.1% for 18S rDNA. In contrast, intraspecific sequence divergences were very low with a maximum sequence difference of 1.0% for *cox1* and 0.2% sequence difference for 16S rDNA sequences among *S. confragosa* individuals. All *S. pinnotherae* individuals had identical *cox1*, 16S, and 18S rDNA sequences (Figs. 3A–3C).

## Discussion

In this study, we identified nine species of Korean rhizocephalans from eight host decapod species using morphological and molecular analyses. Close examination of host ranges revealed that Korean rhizocephalan species have a different host prevalence than reported for rhizocephalan species from other geographic regions. In Korea, rhizocephalans were firstly found from three decapod hosts, *i.e., Hemigrapsus takanoi*, *Macromedaeus distinguendus*, and *Pugettia intermedia*. We also found that most Korean rhizocephalans showed high host specificity, parasitizing only one host, except *Polyascus* cf. *gregarius* that was found on two crab species (Fig. 4). The notable differences in host range between geographic isolates (*i.e.,* rhizocephalans from Korea and other geographic regions) might be due to geographical variation in host species diversity and abundance or insufficient information about the geographic origins of host crab species as proposed by Jung, Yoshida & Kim (2019). In addition, unlike Korean *S. confragosa* individuals that were all found on only one grapsid crab species, *Gaetice depressus*, the Japanese form is known to parasitize three crab species, *G. depressus*, *Pachygrapsus crassipes*, and *Cyclograpsus intermedius*. Furthermore, Japanese *P. yatsui* parasitizes not only *G. depressus*, but also *P. crassipes* (Tsuchida, Lützen & Nishida, 2006; Kobayashi et al., 2018), whereas the Korean form of *P. yatsui* was found only on *Hemigrapsus sanguineus*. We could not determine if other crab species including *P. crassipes* and *C. intermedius* are potential hosts of Korean *S. confragosa* and *P. yatsui* because of the limited pool of crab host species examined in this study. Extensive taxon sampling of decapod hosts and their parasitic barnacles is needed to obtain a complete understanding of the host ranges of rhizocephalan barnacles and the distribution and prevalence of host-parasite associations.

The decapod host infestation rate of Korean rhizocephalan barnacles was much lower than that reported for Japanese species. In Japan, 35 individuals representing three rhizocephalan species were found in 354 individuals of three crab species, corresponding to an infestation rate of 9.9% (Tsuchida, Lützen & Nishida, 2006). By contrast, the infestation rate of Korean rhizocephalans was substantially lower at 1.2% on average (Table 1). Species richness and extent of host usage by parasitic barnacles are tightly correlated to the availability of host species (species diversity and abundance; Kamiya et al., 2014). Differences in the extent of host usage by rhizocephalan barnacles between the two geographic regions are likely due to differences in host species diversity and abundance, as well as the sample size of examined materials (*e.g.*, total numbers of individuals and host species). Since we examined the prevalence of rhizocephalans on all decapod hosts (a total of 3,262 host individuals inspected), our result is likely an accurate estimate of the infestation rate. On the other hand, this prevalence difference between Korea and Japan may be originated from salinity, season, host sex and size (Mouritsen et al., 2018) or biogeographical differences (Kim et al., 2020). In a previous study, the infestation rate of Korean hermit crabs by rhizocephalans was reported to be 0.9% (Jung, Yoshida & Kim, 2019), which is similar to the infestation rate observed in this study. The unexpectedly high infestation rates (>50%) of *Pachygrapsus crassipes* and *Arcotheres sinensis* are due to strong bias from the very small sample size (one to four individuals) examined. The marine ecosystems in different geographic regions display different assemblages of barnacles (Kim et al., 2020) and thus extensive sampling of parasitic barnacles from a wide range of decapod host species is necessary to better understand their prevalence, infection intensity, and host range specificity (Mouritsen et al., 2018).

In addition to their host ranges, morphological and molecular analyses in this study provided insights into the taxonomy of Korean rhizocephalan barnacle species. Phylogenetic trees recognized four monophyletic rhizocephalan families, i.e., Polyascidae, Sacculinidae, Peltogastridae, and Peltogasterellidae, consistent with previous molecular analysis (Høeg et al., 2019) and morphology-based classification. Polyascidae is characterized by multiple externa and reproduces asexually (Glenner, Lützen & Takahashi, 2003), whereas Sacculinidae is characterized by single externa and sexual reproduction. Peltogastridae and Peltogasterellidae species mainly parasitize hermit crabs, and Peltogastridae is distinguished from Peltogasterellidae by the presence of the chitinous shield on its middle part of externae (Høeg et al., 2019). Two new species candidates in the genus *Parasacculina* (*Parasacculina* sp. 1 and *Parasacculina* sp. 2) were recognized based on molecular phylogenetic analyses. These species were distinct from their congeneric species, *P. leptodiae* and *P. sinensis*, based on phylogenetic analyses of mtDNA (16S rDNA) and nuclear (18S rDNA) sequences (Figs. 3B–3C) even though they are morphologically indistinguishable and were found in the same host species. These two species are genetically distinct cryptic species. Furthermore, we transferred three Korean *Sacculina* species (*i.e., Sacculina imberbis*, *S. pilosella*, and *S. pinnotherae*) to the genus *Parasacculina* because they grouped with *Parasacculina* species in mtDNA *cox1*, 16S, and 18S rDNA phylogenetic trees (Figs. 3A–3C). This new taxonomic replacement is consistent with previous studies that transferred several Japanese and Chinese *Sacculina* species to *Parasacculina* based on molecular evidence (Tsuchida, Lützen & Nishida, 2006; Glenner et al., 2010; Høeg et al., 2019).

Comparison of the external cuticles of Korean species with previously published morphological data provided new insight into the taxonomic status of the families Sacculinidae and Polyascidae. Although Høeg et al. (2019) showed that Sacculinidae and Polyascidae are phylogenetically distinct, the original descriptions of Polyascidae (Høeg et al., 2019) did not specify morphological characters differentiating this family from Sacculinidae. For example, Høeg et al. (2019) noted that polyascids have a smooth or almost smooth external cuticle, but some polyascid species (*P. pinnotherae* comb. nov. and *P. yatsui*) in the present study had wrinkled cuticles (Figs. 2C, 2F). In addition, Høeg et al. (2019) mentioned that *Polyascus* species have multiple externae, but *Polyascus* cf. *gregarius* in this study had only a single externa (Fig. 2G). These results indicate that the morphological characteristics of external cuticles, previously considered to be taxonomically valid features, are highly variable and cannot be used as diagnostic characters. Future comparative analyses of morphological characters along with molecular sequences are necessary to confirm the taxonomic status of Sacculinidae and Polyascidae and the taxonomic replacement of the three Korean *Sacculina* species in the genus *Parasacculina.*

## Conclusions

In conclusion, this is the first report of the taxonomy, species diversity, and host ranges of Korean parasitic rhizocephalan barnacles based on morphological and molecular analyses. We identified nine parasitic barnacle species, including two new species candidates in the genus *Parasacculina*, in Korea. In addition, we found higher host specificity and lower infestation rates for Korean rhizocephalan species than reported for rhizocephalan species from other geographic regions. Nevertheless, the results of this study are based on preliminary data derived from limited taxon sampling in a narrow geographic range in Korea. Additional data from extensive samplings of parasitic barnacles from a wide range of crustacean host species are necessary to better understand the taxonomy, prevalence, host usage, and phylogenetic relationships of rhizocephalan species.

## Additional Information and Declarations

### Data Availability

The following information was supplied regarding data availability: Nucleotide sequences are available in GenBank (mtDNA cox1: MZ216468–MZ216513; 16S: MZ215675–MZ215720; 18S rDNA: MZ215557–MZ215602).
